# Intradialytic cycling does not exacerbate microparticles or circulating markers of systemic inflammation in haemodialysis patients

**DOI:** 10.1007/s00421-021-04846-7

**Published:** 2021-12-02

**Authors:** Patrick J. Highton, Daniel S. March, Darren R. Churchward, Charlotte E. Grantham, Hannah M. L. Young, Matthew P. M. Graham-Brown, Seila Estruel, Naomi Martin, Nigel J. Brunskill, Alice C. Smith, James O. Burton, Nicolette C. Bishop

**Affiliations:** 1 School of Sport, Exercise and Health Sciences, Loughborough University, Loughborough, UK; 2 Department of Cardiovascular Sciences, University of Leicester, Leicester, UK; 3 John Walls Renal Unit, Leicester General Hospital, University Hospitals of Leicester, Leicester, UK; 4 Department of Respiratory Sciences, University of Leicester, Leicester, UK; 5 Department of Health Sciences, University of Leicester, Leicester, UK; 6 Department of Physiological Sciences, University of Barcelona, Barcelona, Spain; 7 Leicester School of Allied Health Sciences, Faculty of Health and Life Sciences, De Montfort University, Leicester, UK; 8 Present Address: NIHR Applied Research Collaboration East Midlands, Leicester Diabetes Centre of Research, University of Leicester, Leicester, UK

**Keywords:** Aerobic exercise, End-stage renal disease, Haemodialysis, Microparticles, Intradialytic cycling, Inflammation

## Abstract

**Purpose:**

Patients receiving haemodialysis (HD) display elevated circulating microparticle (MP) concentration, tissue factor (TF) expression and markers of systemic inflammation, though regular intradialytic cycling (IDC) may have a therapeutic effect. This study investigated the impact of regular, moderate-intensity IDC on circulating MPs and inflammatory markers in unit-based HD patients.

**Methods:**

Patients were cluster-randomised to intervention (*n* = 20, age: 51.4 ± 18.1 years, body mass: 77.6 ± 18.3 kg, mean ± SD) or no-exercise control (*n* = 20, 56.8 ± 14.0 years, 80.5 ± 26.5 kg). Intervention participants completed 30 min of moderate intensity (rating of perceived exertion [RPE] of 12–14) IDC, thrice weekly for 6 months. Pre-dialysis venous blood samples were obtained at 0, 3 and 6 months. Circulating MP phenotypes, cytokines, chemokine and MP TF expression were quantified using flow cytometry and cytometric bead array assays.

**Results:**

Despite high exercise compliance (82%), no IDC-dependent effects were observed for any MP, cytokine or chemokine measure (*p* ≥ 0.051, *η*_*ρ*_^2^ ≤ 0.399) other than TNF-α (*p* = 0.001, *η*_*ρ*_^2^ = 0.186), though no significance was revealed upon post hoc analysis.

**Conclusion:**

Six months of regular, moderate-intensity IDC had no effect on MPs, cytokines or chemokines. This suggests that the exercise did not exacerbate thrombotic or inflammatory status, though further functional assays are required to confirm this.

**Trial registration:**

ISRCTN1129707, prospectively registered on 05/03/2015.

## Introduction

People with end-stage kidney disease (ESKD) receiving regular haemodialysis (HD) display elevated cardiovascular mortality, and cardiovascular disease is the leading cause of death in this population (23.3%) (UK Renal Registry 2019). This may be in part due to elevated circulating microparticle (MP) concentrations and altered MP characteristics. MPs are shed upon cellular activation or apoptosis and can act as biomarkers for inflammation and leukocyte dysfunction whilst also promoting thrombosis through tissue factor (TF) expression (Piccin et al., 2007). HD treatment causes increased platelet and neutrophil activation and thus increases MP shedding (Daniel et al. 2006). Furthermore, MPs isolated from HD patients display elevated thrombotic potential compared to the healthy population (Burton et al. 2013) and may be predictive of mortality (Amabile et al. 2011).

HD patients also display chronically elevated circulating concentrations of pro-inflammatory cytokines (e.g. interleukin-6 (IL-6), tumour necrosis factor-alpha (TNF-α)) (Rysz et al. 2006) and reduced circulating concentrations of anti-inflammatory cytokines (e.g. IL-10) (Zhang et al. 2010). Potential causes include chronic uraemia-induced leukocyte ligation, altered leukocyte subset distributions or HD treatment-induced endotoxin exposure (Zhang et al. 2010); (Hauser et al. 2008; Heine et al. 2012). Chronic leukocyte activation also increases the chemokine secretion (e.g. monocyte chemotactic protein-1 (MCP-1)), which positively associates with systemic inflammation (Papayianni et al. 2002) and may drive atherosclerosis in HD patients (Hu et al. 2016).

Both acute aerobic exercise and regular aerobic exercise training can promote favourable changes in MPs (i.e. reduced total and TF + MP concentration) (Babbitt et al. 2013; Highton et al. 2019), an effect which has also been observed in chronic kidney disease and renal transplantation (Highton et al. 2020). In addition, regular aerobic exercise training can reduce circulating pro-inflammatory cytokine and chemokine concentrations (Gleeson et al. 2011; Trøseid et al. 2004), and therefore, may be therapeutic for HD patients. However, HD patients report lack of time and opportunity, as well as safety fears as barriers to traditional exercise participation (Delgado and Johansen 2011). Intradialytic cycling (IDC), which is completed during routine HD treatment under the supervision of exercise professionals or nursing staff, circumnavigates these issues and has been shown to reduce arterial stiffness and cardiovascular risk (Toussaint et al. 2008).

Only one study has investigated the impact of IDC on MPs (Martin et al. 2018), reporting that an acute bout of IDC induced an increase in MP reactive oxygen species release but no change in MP numbers. This is theorised to promote a long-term anti-inflammatory environment through repeated exposure and adaptation, though the long-term effects have yet to be investigated.

An acute bout of moderate-intensity IDC may also prevent an HD-induced increase in IL-6 (Wong et al. 2017) (though non-significantly) or increase circulating IL-10 concentrations to a greater degree than that seen in non-exercising patients (Peres et al. 2015), though some studies investigating regular IDC training have found no impact on inflammatory markers (Dungey et al. 2017; Gołębiowski et al. 2012; Toussaint et al. 2008). However, previous research has exhibited methodological limitations, such as taking resting samples after HD initiation (Dungey et al. 2017), including inappropriate control groups (Gołębiowski et al. 2012) or using small sample sizes (Toussaint et al. 2008). Therefore, there is need for research investigating the impact of regular IDC on MPs and systemic inflammation that addresses these methodological issues. The aim of this study was to investigate how regular, moderate-intensity IDC affects resting MP concentration, TF expression and cytokine and chemokine concentrations.

## Materials and methods

### Ethics and study design

Participants were recruited as a subsample of the ‘CYCLE-HD’ study (ISRCTN1129707), the protocol (Graham-Brown et al. 2016) and primary outcome findings (Graham-Brown et al. 2021) of which have been published previously. Favourable opinion was provided by the NHS Research Ethical Committee (14/EM/1190). Participants were cluster-randomised based on their regular HD shift cohort to usual-care control or intervention groups. Outcome assessments were completed at baseline, 3 and 6 months.

### The intervention

Participants first completed a 1-month familiarisation period, during which they familiarised themselves with the exercise bike and gradually built up their exercise duration to achieve the desired 30 min by the end of this period. Participants then completed 6 months of structured, thrice-weekly moderate-intensity IDC using a cycle ergometer (Letto 2, Motomed, Reck, Germany). Each bout consisted of 30 min of cycling plus 5 min warm-up and cool-down, at an average cadence of 60–70 rpm and a rating of perceived exertion of 12–14. Cycling resistance was progressively increased to maintain RPE in response to exercise adaptation (Graham-Brown et al. 2016). Relevant clinical data, including routine laboratory results, were extracted from medical records.

### Blood sampling

Resting blood samples were taken prior to HD initiation on the second or third HD shift of the week (i.e. after the short inter-dialytic break). 30 mL of venous blood was drawn directly into monovettes containing either K_3_EDTA or sodium citrate (Sarstedt, Nümbrecht, Germany). Sodium citrate-treated blood was centrifuged at 2500 g for 15 min at 20 °C, after which the top 90% was removed and centrifuged again at 2500 g for 15 min at 20 °C—the supernatant (platelet-free plasma) was then removed and stored in 250 µl aliquots at -80 °C for future MP analysis. K_3_EDTA-treated blood was centrifuged at 2500 g for 10 min at 4 °C and the supernatant was separated and stored in 500 µl aliquots at − 80 °C for future cytokine and chemokine analysis.

### Laboratory analysis

**Fig. 1:**
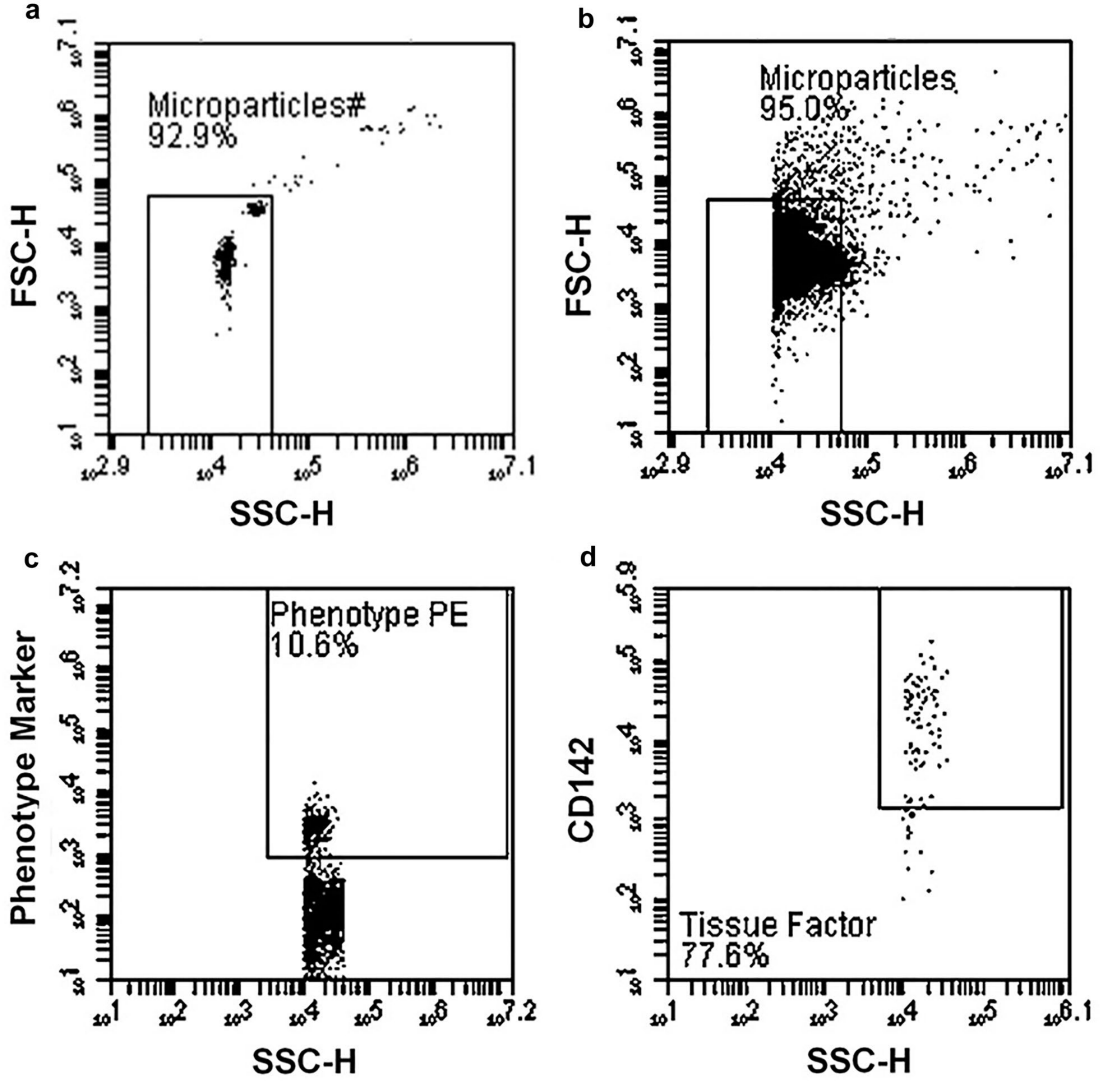
MP flow cytometry gating strategy. **A** Mega-mix beads (BioCytex, Theale, UK, product reference 7801) of known size (0.5 µm, 0.9 µm, and 3.0 µm) to determine subsequent size gating. Two distinct populations (0.5 and 0.9 µm beads, shown in red) are visible, and thus the gate can be set to include these populations whilst excluding the 3.0 µm population (not visible here). **B** ‘All MPs’ based on size, set using previous mega-mix beads. The minimum trigger threshold of 0.3 µm is necessary to exclude the noise floor inherent in all cytometers, hence why the visible MP population (known to be comprised of particles ranging from 0.1 to 1.0 µm in diameter) is cut off at around 10^4^ on the *X* axis. **C** Phenotype marker expression (CD14, CD42b, CD66b, CD144—all four phenotypes employed the same gating strategy), used to quantify MPs of different cellular sources. **D** Tissue Factor expression, back-gated onto the previous graph (**C**) to identify those particles presenting as positive for both their phenotype marker and TF (CD142)

MPs were categorised as platelet-derived (CD42b^+^), neutrophil-derived (CD66b^+^), monocyte-derived (CD14^+^) and endothelial cell-derived (CD144^+^), whilst prothrombotic potential was assessed using TF (CD142^+^) expression. Aliquots of platelet-free plasma were thawed at room temperature prior to analysis. Samples and antibodies were combined and incubated in the dark for 25 min at controlled room temperature (20 °C). The isotype controls used were anti-IgG1k PE, anti-IgG1k APC, anti-IgMk PE and anti-IgG2ak PE. The antibodies used for assessing MPs were anti-AnV FITC, anti-CD42b PE, anti-CD66b PE, anti-CD14 PE, anti-CD144 PE and anti-CD142 APC. Following incubation, samples were analysed using an Accuri C6 flow cytometer for 2 min at a ‘slow’ sampling rate (14 μL·min^−1^) and events were collected into previously established plots (Fig. 1) (CV 13.88%). This methodology and gating strategy was adapted from previous research from our group (Martin et al. 2018) and has been used previously in the general population (Highton et al. 2019) and in those with chronic kidney disease (Highton et al. 2020).

Plasma concentrations of circulating cytokines (IL-2, IL-6, IL-10, IL-17a, and TNF-α) and chemokines (IL-8, MCP-1) were analysed using a cytometric bead array and BD Accuri C6 flow cytometer (BD Biosciences, Oxford, UK). Chemokines were analysed using a pre-configured kit (552990), whilst cytokines were analysed using an enhanced-sensitivity flex-set, combining a master buffer kit (561523) with the corresponding cytokine kits (CV 10.08%). Samples that fell below the minimum detectable threshold (0.274 pg/mL) were assigned a value of 0.01 pg/mL and included in the analyses.

### Statistical analysis

Non-normally distributed data were logarithmically transformed to allow for parametric testing, though all data are presented as non-transformed. Baseline group differences were compared using independent samples t-tests. Exercise progression data were analysed using one-way ANOVAs. The impact of the exercise intervention was assessed using a mixed design ANOVAs. Effect sizes were estimated using partial eta squared (*η*_*ρ*_^2^, 0.02 = small, 0.13 = medium, 0.26 = large) (Bakeman 2005). Upon detection of an interaction effect, independent samples t-tests were completed at each time-point to elucidate the effect. *p* < 0.05 was considered statistically significant. Data are presented as ‘mean ± SD’. Statistical analysis was completed on IBM SPSS version 25.0 (Chicago, Illinois) and graphs were produced on GraphPad Prism (v.7 GraphPad Software Inc., CA, USA).

## Results

### Participants

Baseline demographic and clinical data are presented in Table 1.

**Table 1:** Baseline demographic data

Group	Control (*n* = 20)	Exercise (*n* = 20)
Age (years)	56.8 ± 14.0	51.4 ± 18.1
Male gender, *n*	12	12
Height (m)	1.67 ± 0.08	1.66 ± 0.11
Body mass (kg)	80.49 ± 26.47	77.61 ± 18.27
BMI (kg/m^2^)	28.67 ± 7.53	28.12 ± 6.44
Systolic BP (mmHg)	145 ± 19	142 ± 20
Diastolic BP (mmHg)	72 ± 11	74 ± 13
Dialysis vintage (months)	21.6 ± 19.3	33.0 ± 27.1
Hypertension (*n*, %)	16 (80)	15 (75)
Diabetes (*n*, %)	9 (45)	5 (25)

Where appropriate, data are presented as ‘mean ± SD’

### Exercise sessions

In total, 84% of all IDC sessions were completed (± 13%, range 48–99%). The mean gear (indicative of intensity) achieved during each session increased from baseline to months five (*p* = 0.026) and six (*p* = 0.024). No other exercise session characteristics changed throughout the programme (data not shown).

### Circulating microparticles

**Fig. 2:**
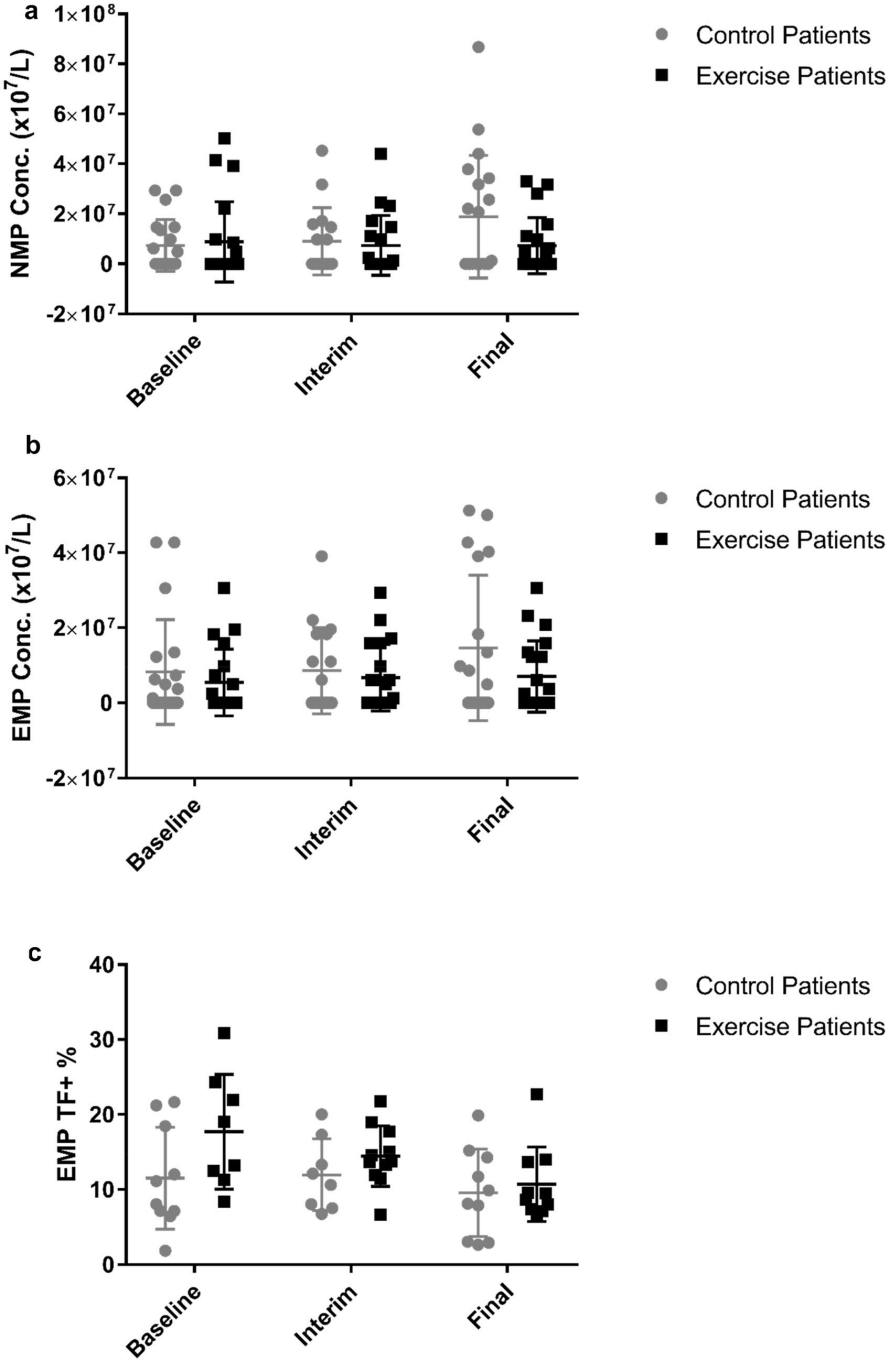
Circulating concentration of neutrophil-derived MPs (NMP) (**a**), endothelial cell-derived MPs (EMP) (**b**) and the percentage of EMPs that express TF (**c**). Data are presented as ‘mean ± SD’. Concentration data were non-normally distributed and thus logarithmically transformed prior to analysis, whilst percentage data were normally distributed (control *n* = 20, exercise *n* = 20)

MP results are presented in Fig. 2 and Table 2. Both total MPs and PS/Annexin-V^+^ MPs were similar at baseline with no evidence of IDC-dependent effects (*p* ≥ 0.326, *η*_*ρ*_^2^ ≤ 0.009). There was also no evidence for IDC-dependent effects observed in the circulating concentration of total platelet-derived MPs or TF^+^ platelet-derived MPs, or the % of total MPs that were platelet-derived or platelet-derived MPs that were TF^+^ (*p* ≥ 0.279, *η*_*ρ*_^2^ ≤ 0.035).

**Table 2:** MP phenotype and TF expression data

	Control (*n* = 20)			Exercise (*n* = 20)		
	Baseline	Interim	Final	Baseline	Interim	Final
Total MP conc. (× 10^9^/L)#	12.30 ± 10.96	12.26 ± 18.29	17.96 ± 23.57	14.76 ± 17.84	22.29 ± 29.01	20.71 ± 20.08
AnV^+^ MP conc. (× 10^7^/L)#	13.59 ± 9.30	9.08 ± 7.02	17.03 ± 12.25	11.15 ± 7.20	10.56 ± 7.20	12.72 ± 6.98
PMP conc. (× 10^7^/L)#	7.91 ± 4.52	6.33 ± 7.56	8.48 ± 5.63	9.19 ± 3.98	10.21 ± 7.87	8.87 ± 6.08
TF^+^ PMP conc. (× 10^7^/L)	2.26 ± 1.43	1.72 ± 1.61	1.98 ± 1.57	2.69 ± 1.65	2.52 ± 1.30	2.41 ± 1.52
% PMP of total MPs	1.59 ± 1.92	0.85 ± 1.03	1.75 ± 2.59	1.99 ± 2.73	1.42 ± 1.61	1.84 ± 2.70
% PMP TF^+^	28.46 ± 14.40	29.60 ± 15.52	23.70 ± 12.25	29.83 ± 13.91	32.30 ± 12.79	31.08 ± 11.98
TF^+^ NMP conc. (× 10^7^/L)#	0.13 ± 0.17	0.24 ± 0.40	0.20 ± 0.31	0.17 ± 0.27	0.11 ± 0.18	0.15 ± 0.22
% NMP of total MPs	0.24 ± 0.49	0.08 ± 0.13	0.20 ± 0.36	0.21 ± 0.54	0.07 ± 0.13	0.11 ± 0.27
% NMP TF^+^	19.84 ± 12.25	23.57 ± 11.05	18.90 ± 8.77	23.78 ± 10.69	26.21 ± 10.29	23.60 ± 5.99
MMP conc. (× 10^7^/L)#	3.82 ± 3.04	3.78 ± 3.53	3.91 ± 3.35	5.03 ± 3.53	4.21 ± 3.49	4.00 ± 3.94
TF^+^ MMP conc. (× 10^7^/L)#	0.84 ± 0.76	0.73 ± 0.80	0.86 ± 0.89	1.24 ± 0.98	0.85 ± 0.67	0.97 ± 1.34
% MMP of total MPs	0.81 ± 1.57	0.51 ± 0.80	1.14 ± 2.15	0.97 ± 1.48	0.72 ± 0.81	1.09 ± 1.92
% MMP TF^+^	21.16 ± 7.60	18.92 ± 6.75	20.56 ± 9.97	24.14 ± 10.00	20.92 ± 8.54	23.90 ± 6.39
TF^+^ EMP conc. (× 10^7^/L)#	0.06 ± 0.13	0.09 ± 0.13	0.05 ± 0.09	0.06 ± 0.09	0.10 ± 0.13	0.08 ± 0.09
% EMP of total MPs	0.10 ± 0.18	0.16 ± 0.31	0.19 ± 0.45	0.08 ± 0.18	0.26 ± 0.40	0.32 ± 0.63

All data are presented as ‘mean ± SD’

*AnV* Annexin-V; *PMP* platelet-derived MP; *NMP* neutrophil-derived MP; *MMP* monocyte-derived MP; *EMP* endothelial cell-derived MP; *TF* tissue factor

#Indicates that data were non-normally distributed and thus were logarithmically transformed prior to analysis

The number of neutrophil-derived MPs was similar in both groups at baseline (*p* = 0.279). Although there was an increase in the number of neutrophil-derived MPs over time in the control group, there was insufficient evidence overall (time*group interaction effect *p* = 0.051, *η*_*ρ*_^2^ = 0.096) to demonstrate a difference between exercise and control groups after 6 months (*p* = 0.063) (Fig. 2a). In addition, no other neutrophil-derived MP variable (TF^+^ neutrophil-derived MPs, % of total MPs that were neutrophil-derived MPs, % of neutrophil-derived MPs that were TF^+^) displayed evidence of time*group interaction effects (*p* ≥ 0.261, *η*_*ρ*_^2^ ≤ 0.040).

Similarly, the circulating concentration of total and TF^+^ monocyte or endothelial-derived MPs, the % of total MPs that were monocyte or endothelial-derived and the % of monocyte or endothelial-derived MPs that were TF^+^ all showed no evidence of any baseline differences (*p* ≥ 0.343) or time*group interaction effects (*p* ≥ 0.097, *η*_*ρ*_^2^ ≤ 0.399) (Fig. 2b, c).

### Circulating cytokines and chemokines

**Fig. 3:**
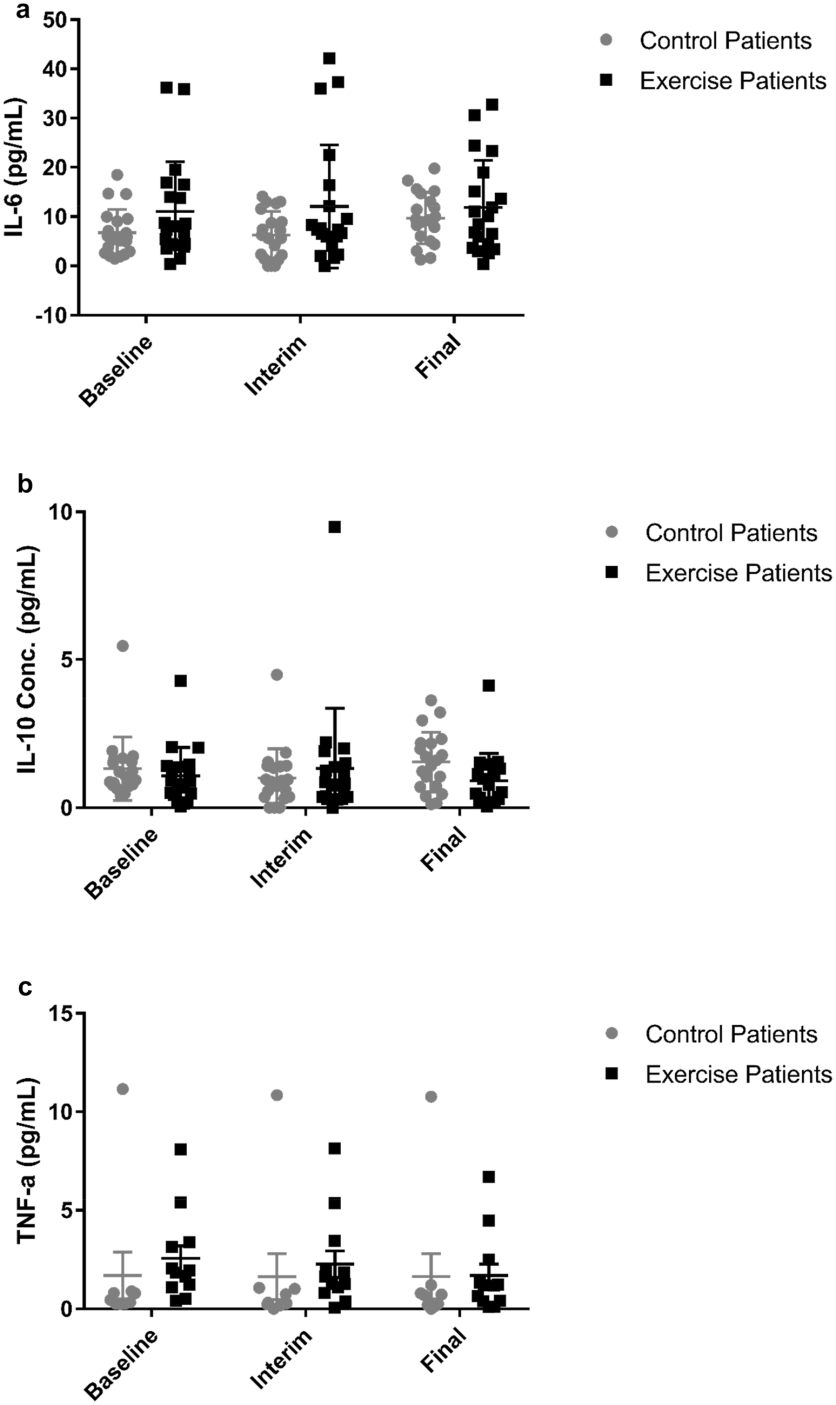
Circulating IL-6 (**a**), IL-10 (**b**) and TNF-α (**c**) levels. Data are presented as ‘mean ± SD’. These data were non-normally distributed and thus logarithmically transformed prior to analysis (control *n* = 20, IDC exercise *n* = 20)

All IL-2 values fell below the minimum detectable threshold and so analysis was not completed. All samples (*n* = 40) provided detectable IL-6 and IL-10 values (Fig. 3a, b), though there was insufficient evidence to suggest the presence of baseline differences or interaction effects (*p* ≥ 0.060, *η*_*ρ*_^2^ ≤ 0.076).

Two control and seven IDC participants displayed IL-17a results above the minimum detectable threshold—the remainder were assigned as 0.01 pg/mL. No baseline group differences or interaction effects were observed (*p* ≥ 0.262, *η*_*ρ*_^2^ ≤ 0.150).

TNF-α values above the minimum detectable threshold were observed in 9 control participants and 12 IDC participants, whilst the remaining values were assigned as 0.01 pg/mL. No baseline group differences were observed (*p* = 0.294), though there was strong evidence to suggest a significant time*group interaction effect (*p* = 0.001, *η*_*ρ*_^2^ = 0.186). However, no further statistical significance was detected upon post hoc analysis (Fig. 3c).

Circulating concentrations of IL-8 and MCP-1 displayed no evidence of baseline differences (*p* ≥ 0.101) or time*group interaction effects (*p* ≥ 0.151, *η*_*ρ*_^2^ ≤ 0.053) (Table 3).

**Table 3:** Circulating chemokine data

Chemokine	Control (*n* = 20)			Exercise (*n* = 20)		
	Baseline	Interim	Final	Baseline	Interim	Final
IL-8 (pg/mL)	23.70 ± 15.60	20.09 ± 13.90	21.71 ± 17.80	20.68 ± 9.61	20.00 ± 10.51	21.41 ± 11.67
MCP-1 (pg/mL)	47.06 ± 23.47	35.30 ± 10.20	44.64 ± 18.25	39.81 ± 25.31	39.65 ± 22.94	41.30 ± 24.55

Data are presented as ‘mean ± SD’. These data were normally distributed

### Clinical laboratory outcomes

No evidence was observed to suggest that the intervention had any effects on relevant clinical results obtained from medical records (Table 4).

**Table 4:** Clinical laboratory results obtained from medical records at baseline, interim and final time-points

	Control (*n* = 20)			Exercise (*n* = 20)		
	Baseline	Interim	Final	Baseline	Interim	Final
White cell count (× 10^9^/L)	7.86 ± 2.54	7.03 ± 1.97	7.67 ± 2.50	6.84 ± 2.16	6.64 ± 1.57	6.88 ± 1.96
Neutrophils (× 10^9^/L)	4.43 ± 1.37	4.58 ± 1.18	4.99 ± 1.51	4.22 ± 1.71	4.39 ± 1.48	4.12 ± 1.04
Lymphocytes (× 10^9^/L)	1.8 ± 0.5	1.68 ± 0.60	1.61 ± 0.60	1.7 ± 0.8	1.48 ± 0.69	1.73 ± 0.61
Monocytes (× 10^9^/L)	0.5 ± 0.2	0.54 ± 0.09	0.51 ± 0.17	0.5 ± 0.2	0.48 ± 0.15	0.45 ± 0.15
Total cholesterol (mmol/L)	3.93 ± 0.98	4.08 ± 1.08	3.98 ± 1.17	4.29 ± 1.25	4.29 ± 1.70	4.39 ± 1.49
Triglycerides (mmol/L)	1.85 ± 1.50	1.90 ± 1.24	1.64 ± 0.98	1.65 ± 1.02	1.76 ± 1.28	1.74 ± 0.71
Urea reduction ratio (%)	74 ± 14	78 ± 6	76 ± 6	77 ± 8	75 ± 13	75 ± 9

No significant exercise-dependent effects were observed. All data were normally distributed and are presented as ‘mean ± SD’

## Discussion

This study found no evidence to suggest that a 6-month programme of thrice-weekly structured IDC training exacerbates circulating MP, cytokine, or chemokine concentrations or MP tissue factor expression.

Whilst a reduction in neutrophil-derived MP concentration was observed in the IDC group, thus suggesting a potential anti-inflammatory effect of regular IDC, there was insufficient evidence (*p* = 0.051) to firmly support this result, and therefore, this finding should be considered hypothesis-generating only. If future research reveals stronger evidence for this effect, possible mechanistic explanations that could be investigated include reduced uraemia-induced neutrophil activation or reduced neutrophil apoptosis resulting in reduced neutrophil-derived MP shedding. However, whilst the HD procedure can drive leukocyte apoptosis (Moser et al. 2003), the impact of IDC has not yet been investigated. As HD patients commonly experience impaired circulation (McIntyre et al. 2008), regular IDC training may restore systemic circulation and prevent hypoxia, reducing neutrophil activation. A reduction in circulating neutrophil-derived MPs would be expected to represent an anti-inflammatory effect, which could ultimately reduce thrombosis and cardiovascular risk, however, the impact of IDC in this regard can only be postulated based on the findings from this study.

Circulating concentrations of MPs derived from endothelial cells, platelets and monocytes are increased in patients with renal failure, and associate with vascular dysfunction (Daniel et al. 2008). The MP results of this study mimic those investigating acute IDC (Martin et al. 2018), and as such it may be that the exercise was not of a sufficient intensity in either case to elicit significant reductions in circulating MP concentrations, either acutely or on a long-term basis. However, as the mean power outputs achieved in this study were similar to those of previous IDC research (~ 21–22 W) (Dungey et al. 2015; Martin et al. 2018), this exercise intensity is likely to be representative of what can be reasonably expected of HD patients. As such, whilst regular aerobic exercise has been previously reported to elicit MP-mediated health benefits in other populations (Highton et al. 2018), there is as yet insufficient evidence to suggest that this can be achieved with regular IDC.

Supporting this, whilst previous research investigating acute IDC has suggested the possibility of an MP-mediated anti-inflammatory effect of long-term IDC (Martin et al. 2018), this was not clearly observed here. Therefore, it may be that any possible transient effect of acute moderate-intensity IDC on MPs is not sufficient to induce a negative feedback loop potent enough to elicit an overall anti-inflammatory effect. In addition, whilst previous research has displayed beneficial effects of regular moderate-intensity aerobic exercise on MP concentrations in the general population (Babbitt et al. 2013), this also was not observed here in this population of HD patients. This may be due in part to discrepancies in exercise intensity—Babbitt et al. incorporated objectively measured exercise intensity training (65% of VO_2max_) whilst here it was subjective (RPE 12–14), which may have resulted in a, respectively, lower achieved intensity. It may also be that the potent effect of end-stage renal failure and the HD procedure on MPs (Amabile et al. 2011; Daniel et al. 2006) prevents the observation of any effects induced by exercise of this intensity. However, there is currently no evidence to suggest that regular IDC exacerbates MP status in HD patients, though further functional assays would be required to investigate this more directly, for instance a prothrombinase assay to assess prothrombotic potential.

As there was insufficient evidence here to suggest the presence of ad MP-mediated anti-inflammatory response, it is unsurprising that no IDC-dependent effects were observed in circulating cytokines. The diverse battery of cytokines assessed here typically exhibit either pro-inflammatory effects (TNF-α, IL-17a), anti-inflammatory effects (IL-2, IL-10), or both (IL-6) (Berg and Scherer 2005; Cortvrindt et al. 2017; Hoyer et al. 2008; Stenvinkel et al. 2005), though there was no evidence to suggest any exercise-dependent effects, either pro- or anti-inflammatory, despite initial evidence suggesting a reduction in TNF-α. In previous research in the general population, both acute and regular aerobic exercise participation have elicited favourable changes in these cytokines (Gleeson et al. 2011; Rhind et al. 1996), and similar anti-inflammatory effects have also been displayed following acute IDC (Peres et al. 2015). A previous study investigating regular IDC also found no observable change in circulating cytokines despite observing reductions in circulating intermediate monocyte proportion, though at a slightly lower exercise intensity (16 W) (Dungey et al. 2017). It may be that HD patients cannot routinely achieve an exercise intensity sufficient enough to impact their resting systemic inflammatory profile. MCP-1 and IL-8, pro-inflammatory chemokines that promote leukocyte migration and associate with atherosclerosis and cardiovascular risk (Hashmi and Zeng 2006; Kusano et al. 2004; Papayianni et al. 2002), also displayed no IDC-dependent effects, likely for the same reason. However, it is encouraging that these circulating mediators were not exacerbated by regular IDC, which points towards the ‘immunological safety’ of this form of exercise in this population.

Flow cytometry is considered the gold-standard technique most applicable to clinical research for the detection and characterisation of MPs due to its high throughput and the level of information it can provide concerning MP size, complexity and surface marker expression (Strasser et al. 2013; Van der Pol et al. 2014). In addition, flow cytometry allows for several steps of confirmation when detecting and measuring MPs, including confirmation based on size, internal complexity, and the expression of one or a combination of specific markers, which makes it easier to isolate the population of interest. Whilst this is possible with other techniques, such as nanoparticle-tracking analysis, it is more arduous and less comprehensive. Nanoparticle tracking analysis does have the advantage over traditional flow cytometry of being able to visualise the particles of interest (though this is possible with imaging flow cytometry (Lannigan and Erdbruegger 2017)). In addition, nanoparticle-tracking analysis typically provides a lower minimum detectable threshold (0.05 µm) than this flow cytometry technique (0.3 µm) (Highton et al. 2019), which is a previously identified limitation of flow cytometry (Burton et al. 2013). Therefore, an accepted limitation of the flow cytometry technique employed here is that it may be excluding a certain number of MPs below 0.3 µm in diameter; however, it could also be argued that using nanoparticle-tracking analysis could also incorporate unwanted exosomes in the 0.05–0.1 µm range in the analyses. Ideally, methodologies for isolating and analysing MP populations would employ two or more techniques to capitalise on the advantages of each technique and to ensure that the full size range is captured (Strasser et al. 2013; Szatanek et al. 2017). However, this was not possible in this study due to resource constraints, and therefore, flow cytometry was selected due to its advantages over other techniques (Szatanek et al. 2017). Further research should focus on harmonising the various techniques for MP detection to construct a comprehensive, standardised methodology.

### Limitations

This study incorporated only a cohort of the full CYCLE-HD study and as such was not informed by an a priori power calculation, though the sample size included was much larger than the majority of IDC studies that have investigated MPs or systemic inflammation in detail in the current body of literature. In addition, only one technique was employed for MP analysis which may have limited the range of captured particles, though this was due to the resource demand of employing more than one laboratory analysis technique. Lastly, the cytometric bead array technique was unable to detect certain cytokines (IL-2 and IL-17a) in a large number of samples suggesting insufficient assay sensitivity, though as IL-6 and IL-10 were detected above the minimum threshold in every sample, it is likely that the concentrations were simply too low.

### Conclusions and future research

In conclusion, this study found no evidence to suggest that a 6-month programme of thrice-weekly intradialytic cycling training altered circulating MP, cytokine or chemokine concentrations or MP tissue factor expression. Whilst this suggests the immunological safety of IDC, these findings can only be considered as hypothesis generating and should be pursued, primarily by incorporating further functional assays to investigate the impact on thrombotic potential and immune function. In addition, future research should aim to investigate the feasibility of methods to achieve greater exercise intensity using IDC, and whether this impacts MPs or the inflammatory environment in HD patients.

## Data Availability

Data are available from the corresponding author upon reasonable request.

## Abbreviations

ANOVA: Analysis of variance
CD: Cluster of differentiation
CV: Coefficient of variation
ESKD: End-stage kidney disease
HD: Haemodialysis
IDC: Intradialytic cycling
IL: Interleukin
MCP: Monocyte chemotactic protein
MP: Microparticle
RPE: Rate of perceived exertion
SD: Standard deviation
SPSS: Statistical package for the social sciences
TF: Tissue factor
TNF-α: Tumour necrosis factor-alpha
